# Blestriarene C exerts an inhibitory effect on triple-negative breast cancer through multiple signaling pathways

**DOI:** 10.3389/fphar.2024.1434812

**Published:** 2024-10-22

**Authors:** Junsha An, Mingyu Han, Hailin Tang, Cheng Peng, Wei Huang, Fu Peng

**Affiliations:** ^1^ West China School of Pharmacy, Sichuan University, Chengdu, China; ^2^ State Key Laboratory of Southwestern Chinese Medicine Resources, Chengdu University of Traditional Chinese Medicine, Chengdu, China; ^3^ State Key Laboratory of Oncology in South China, Guangdong Provincial Clinical Research Center for Cancer, Sun Yat-Sen University Cancer Center, Guangzhou, China; ^4^ Key Laboratory of Drug-Targeting and Drug Delivery System of the Education Ministry, Sichuan Engineering Laboratory for Plant-Sourced Drug and Sichuan Research Center for Drug Precision Industrial Technology, Sichuan University, Chengdu, China

**Keywords:** blestriarene C, triple-negative breast cancer, network pharmacology, HSP90AA1, PTGS2

## Abstract

**Introduction:**

Breast cancer is the most common cancer worldwide, the leading cause of cancer death in women, and the fifth leading cause of cancer death. Triple negative breast cancer (TNBC), with high metastasis and mortality rates, is the most challenging subtype in breast cancer treatment. There is an urgent need to develop anti-TNBC drugs with significant efficacy, low side effects and good availability. In early drug screening, blestriarene C was found to have inhibitory effects on TNBC cells. In this article, we further explore the mechanisms associated with blestriarene C for breast cancer.

**Methods:**

In this article, we take the approach of network pharmacology combined with in vivo and in vitro experiments. Network pharmacology analysis was used to predict the active components in Baiji, and to investigate the hub targets and related mechanisms of BC in TNBC treatment. The mechanism of anti-TNBC in vitro was evaluated by CCK-8 assay, cell apoptosis and cell cycle assays, wound healing assay, WB assay, and molecular docking analysis. The inhibition effect in vivo was test in subcutaneous tumor models established in mice.

**Results:**

Through network pharmacology analysis and experiments, we screened out BC as the main active ingredient, and found that BC could inhibit the Ras/ERK/c-Fos signaling pathway while downregulating the expression of HSP90AA1 and upregulating the expression of PTGS2, thereby promoting apoptosis, causing S-phase cycle arrest, and inhibiting the proliferation and migration of BT549 cells. The *in vivo* results illustrated that BC inhibited the growth of TNBC tumors and has a high safety profile. By integrating network pharmacology with *in vitro* and *in vivo* experiments, this study demonstrated that BC inhibited the proliferation and migration of TNBC cells by inhibiting the Ras/ERK/c-Fos signaling pathway, promoting apoptosis, and causing S-phase cycle arrest.

**Discussion:**

This study provides new evidence for the use of BC as a novel drug for TNBC treatment.

## 1 Introduction

Breast cancer is a phenomenon in which breast epithelial cells proliferate out of control under the action of a variety of oncogenic factors. Among women, breast cancer is projected to be the most common tumor and second only to lung cancer in the number of deaths from new cases (Siegel et al., 2024). A study published in 2022 not only provides a global overview of breast cancer incidence in 2020, but also estimates its trend in 2040, with more than three million new cases and more than one million deaths per year, with an increase in morbidity and mortality of 40% and 50%, respectively (Arnold et al., 2022), and overcoming breast cancer has become an increasingly global concern.

Triple-negative breast cancer (TNBC) is an aggressive breast cancer subtype that is devoid of the expression of the estrogen receptor (ER), progesterone receptor (PR) and human epidermal growth factor receptor 2 (HER2) (Cortes et al., 2022; Jia et al., 2017). Compared with other types of breast cancer, TNBC has the characteristics of strong aggressiveness, high risk of metastasis, and poor clinical prognosis (Bianchini et al., 2016). Cytotoxic chemotherapy remains the common treatment for breast cancer, with the backbone consisting of carboplatin plus paclitaxel followed by anthracycline and cyclophosphamide (Leon-Ferre and Goetz, 2023). For the HER2-enriched subtype, trastuzumab is generally added to neoadjuvant chemotherapy regimens, and satisfactory results have been obtained in clinical use. Although the efficacy of neoadjuvant chemotherapy has been proven, clinical trial data show that the effect of neoadjuvant chemotherapy varies greatly among different breast cancer patients, and it is prone to drug resistance, which is not conducive to subsequent treatment (Schettini et al., 2020; Shen et al., 2021). There is an urgent need to develop anti-TNBC drugs with significant efficacy and low side effects.

*Bletilla striata* (Thunb. ex Murray) Rchb. F. (Baiji in Chinese), a plant in the family Orchidaceae, is found in China, Japan, the Korean Peninsula, and other countries. Among them, it is widespread in the Yangtze River Basin provinces in China and rich in resources. Moreover, it is a traditional Chinese medicine that is widely used in China and has a long history, having first been mentioned in the *Shennong herbal Scripture*. Baiji has a variety of pharmacological activities, such as antibacterial, anti-inflammatory, antioxidant, immunomodulation, anti-tumor, anti-aging, etc., and has a high degree of safety (He et al., 2017). Several types of chemical constituents have been studied in its extracts, including biphenyls, phenanthrenes, dihydrophenanthrenes, biphenanthrenes, and quinone derivatives.

**FIGURE 1:**
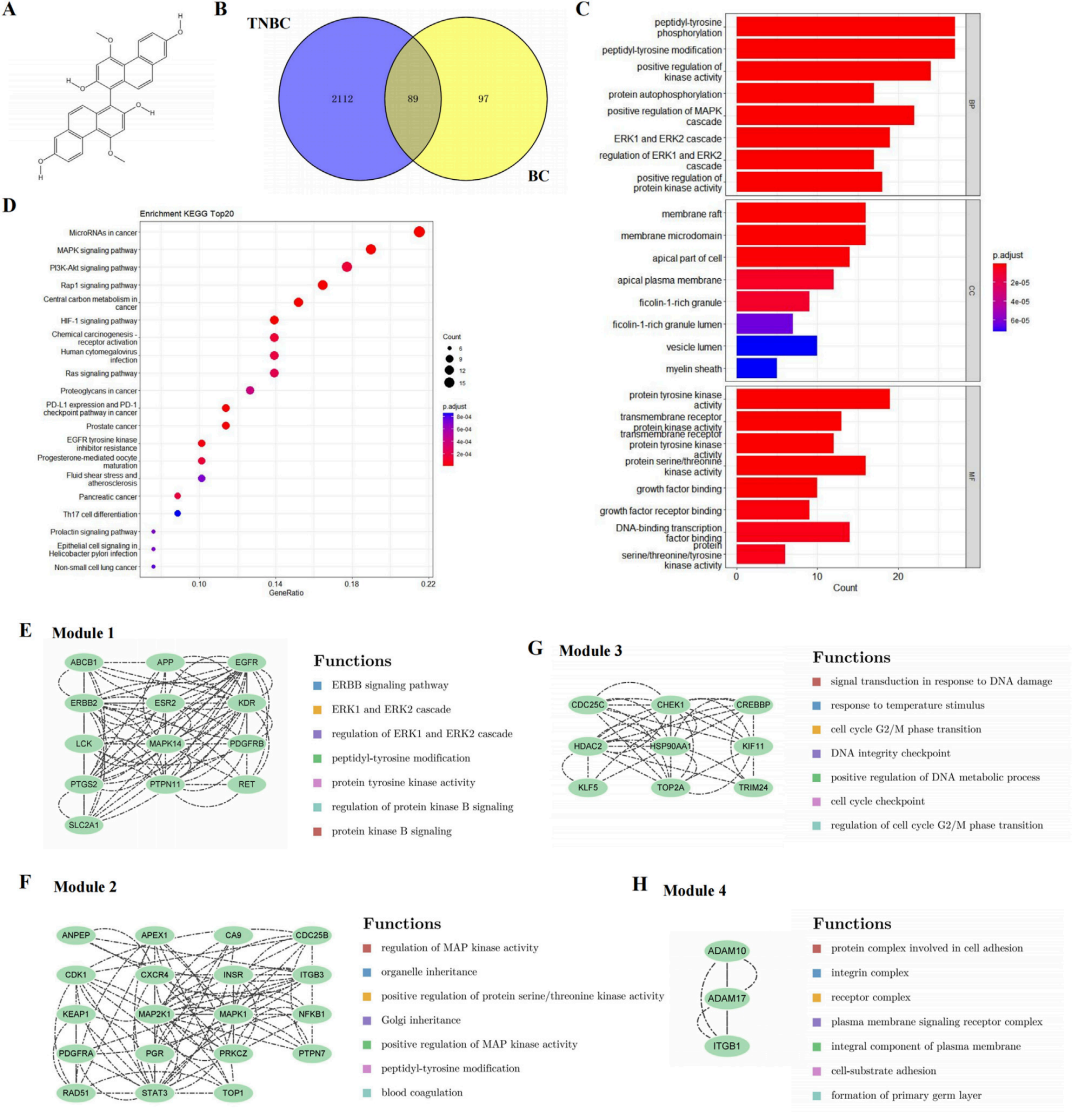
Candidate targets and their functional enrichment analysis. **(A)** Chemical structure of BC. **(B)** Candidate targets of BC in TNBC treatment. **(C)** KEGG pathway enrichment analysis (top 20). **(D)** GO enrichment analysis, including the top 10 significant enrichment terms of BP, CC and MF. **(E–H)** Four significant gene clustering modules and their functions.

Blestriarene C (BC), whose structure is shown in Figure 1A, is a dibiphenine compound extracted from Baiji. The literature suggests that similar compounds have good tumor inhibitory effects and no serious side effects, suggesting that BC has the potential to be used as a novel treatment for TNBC (Kovács et al., 2008; Li et al., 2018).

This paper used network pharmacological prediction and experimental verification to explore the inhibitory effect of BC, an active ingredient of Baiji, on TNBC, tentatively reveal its mechanism of action, so as to provide a reference for the development of drugs for TNBC treatment.

## 2 Materials and methods

### 2.1 Network pharmacology research

#### 2.1.1 Screening of active compounds in Baiji and prediction of candidate targets

The TCMSP database was used to get all active compounds of Baiji, and our filter was set to “Oral Bioavailability (OB) > 30%” and “Drug-likeness (DL) > 0.18”. We collected all the active compounds of Baiji, and screened out BC as the main active ingredient.

The SuperPred database and the SwissTargetPrediction database were used to obtain potential targets of BC by mapping its structure. Then, targets from different databases were combined, duplicates were removed, and candidate targets were obtained.

We searched for TNBC-related targets using “triple-negative breast carcinoma” and “triple negative breast cancer” as keywords in the DisGeNet database and the GeneCards database. “Relevance score ≥10” was used as a filter. The results of the two were then combined and duplicate targets were removed.

The Venny 2.1.0 was used to screen out the intersection of potential targets of BC and TNBC-related targets.

#### 2.1.2 Functional annotation and pathway enrichment analysis

To further reveal the functions of the candidate targets, GO and KEGG enrichment analysis were performed and visualized by the “clusterProfiler 4.0” package using R software (Wu et al., 2021). *P*< 0.05 was set as the cutoff criterion.

#### 2.1.3 PPI network construction and module analysis

All the targets were uploaded to the String database to construct the PPI network interaction. Cytoscape 3.9.1 was used to construct and visualize the PPI network. MCODE, a plug-in for Cytoscape, was used to analyze key functional modules. The criteria were set as follows: K-core = 2, degree cutoff = 2, node score cutoff = 0.2, and max depth = 100. What’s more, we analyzed related functions of the key functional modules based on the GeneMANIA database.

#### 2.1.4 Screening of hub genes

CytoHubba, a network topology analysis plug-in in Cytoscape, was used to analyze the topology parameters of each target. Eight common algorithms (MCC, MNC, EPC, Degree, Closeness, Radiality, Betweenness, Stress) were used to evaluate and select hub genes.

Additionally, we used the GSCA database to perform gene expression validation and survival analysis on hub genes.

#### 2.1.5 Screening of key microRNAs

The miRTarBase database contains the experimentally validated microRNA-target interactions. In it, we searched for microRNAs corresponding to core targets whose validation methods had strong evidence (reporter assay, Western blot, or qPCR) and whose strong evidence methods had greater than or equal to two. Then, the interaction network diagram of “core target-microRNA” was plotted in Cytoscape 3.9.1. The top five microRNAs were further screened as the most functional microRNAs, sorted by degree value.

In addition, we performed miRNA expression and Kaplan-Meier survival analysis on the screened microRNA in the CancerMIRHome database, and the selected conditions were TCGA database and BRCA for tumors.

#### 2.1.6 Analysis of expression and prognostic value of selected proteins

We examined the expression of HSP90AA1 and PTGS2 in BRCA, based on the TCGA and GTEx databases. We then validated HSP90AA1 and PTGS2 protein expression in the HPA database.

#### 2.1.7 Analysis of mutation and methylation of selected proteins

The cBioPortal database was used to collect information on the alteration frequency, mutation types, and mutation sites of HSP90AA1 and PTGS2. The Ualcan database was used to analyze promoter methylation. In addition, we integrated the mutation data and gene expression data, and analyzed the effects of CNV and SNV on gene expression. Finally, we analyzed the expression of 44 marker genes of three types of RNA modification (m1A, m5C, m6A), and calculated the correlation between HSP90AA1, PTGS2 and methylation.

### 2.2 Experimental validation

#### 2.2.1 Reagents

BC was identified and provided by Professor Wei Huang of Chengdu University of Traditional Chinese Medicine.

RPMI Medium 1,640, 10% Fetal Bovine Serum (FBS), and 0.25% Trypsin-EDTA were purchased from Gibco (NY, United States). Cell Counting Kit-8 was bought from Dojindo (Kumamoto, Japan). Annexin V-FITC/PI Apoptosis Detection Kit, the primary antibodies against HSP90AA1, PTGS2, and GAPDH were purchased from BOSTER (California, United States). The primary antibodies against c-Fos, ERK1/2, phospho-ERK1/2, Cyclin A2, CDK2, Bcl-2, Bax, Cleaved Caspase-3, Survivin, p53, and HSP90 were bought from Cell Signaling Technology (Boston, United States). Penicillin-Streptomycin Solution was obtained from Cytiva (Shanghai, China). Cell Cycle and Apoptosis Analysis Kit, BCA Protein Quantitation Kit, SDS-PAGE Sample Loading Buffer, Precast PAGE Gel, SDS-PAGE Hepes Electrophoresis Buffer were purchased from Beyotime (Shanghai, China). EveryBlot Blocking Buffer was obtained from BIO-RAD (California, United States). The primary antibodies against CDK1 was bought from Abcam (Cambridge, United Kingdom).

#### 2.2.2 Cell culture

BT549 cells were obtained from Procell (Wuhan, China). They were cultured in RPMI 1640 medium supplemented with 10% (v/v) FBS and 1% (v/v) penicillin-streptomycin solution, and maintained at 37°C in a humidified chamber with 5% CO_2_.

#### 2.2.3 Cell viability assay

Cell viability was measured using CCK-8 assay. BT549 cells were seeded in 96-well plates and exposed to BC (0, 2.5, 5, 7.5, 10, 15, 20, 30 μmol. L^−1^), and Z-VAD-FMK, an apoptosis inhibitor, in combination with BC for 24 h. Then, 10 μL of CCK-8 solution was added to each well of the plate. After 1 h of incubating, we measured the optical density (OD) at 450 nm wavelength with a microplate reader. We also detected the inhibition rate of low concentrations of BC (0, 0.125, 0.25, 0.5, 1, 2.5, 5, 10, 20 μmol. L^−1^). (Cell viability = [(OD_Experiment group_ - OD_Blank_)/(OD_Control group_ - OD_Blank_)] × 100%).

#### 2.2.4 Cell apoptosis and cell cycle assays

Cell apoptosis and cell cycle were analyzed by annexin V-FITC/PI apoptosis detection kit and cell cycle and apoptosis analysis kit with a FACSCalibur flow cytometer. BT549 cells were treated with different concentrations of BC (0, 2.5, 5, and 10 μmol/L) for 24 h, and cell apoptosis and cell cycle were detected according to the instructions.

#### 2.2.5 Wound healing assay

The concentration with no inhibitory effect on the proliferation of BT549 cells was selected to detect the migration inhibitory effect of BC by wound healing assay. BT549 cells were seeded into 6-well plates. Scratching wounds with a pipette tip when the cell density reached 90%. Then different concentrations of BC (0, 0.025, 0.05, 0.1 μmol. L^−1^) prepared in 1% FBS medium were added to the cells of the corresponding groups. Images were taken by microscopy at 0, 6, 12 and 24 h to evaluate the ability of cell migration. (Cell migration rate = [(initial scratch area - scratch area at moment t)/initial scratch area] × 100%).

#### 2.2.6 Western blotting assay

BT549 cells were seeded into cell culture dishes and exposed to BC for 24 h. After extracting protein, electrophoresis, and transferring according to the procedure as directed, we used the primary antibodies overnight at 4°C. The protein bands were detected by the CheniDoc MP Imaging System.

#### 2.2.7 Molecular docking between BC and selected proteins

The PubChem database was used to download the SDF format of BC. The 3D structure of HSP90AA1 and PTGS2 proteins was downloaded from the PDB database.

Then the search results were imported into the AutoDock Tool software, and the binding conformation with the lowest free binding energy was selected by using AutoDock Vina and imported into PyMol software for visualization.

#### 2.2.8 Cellular thermal shift assay (CETSA)–Western bloting (WB)

BT549 cells were seeded into cell culture dished and treated with BC (0 and 5 μmol L^−1^) for 24 h. BT549 cell precipitates were collected and heated at 10 temperature points (37, 41, 44, 47, 50, 53, 56, 59, 63, 67°C) for 3 min, followed by repeated freezing and thawing of the cells by liquid nitrogen for 2 times to lysed the cells, and the supernatant was collected. Finally, the experiment was performed with reference to the steps of WB assay.

#### 2.2.9 Animals and treatment

7-week-old specific pathogen-free (SPF) female BALB/c-nu mice were purchased from Dossy (Chengdu, China). Mice were randomly divided into two groups (n = 8) for different experimental analyses. Mice in the experimental group were given intraperitoneal injection (i.p.) of BC (1 mg/kg/d), while mice in the control group received an equal volume of normal saline. The treatment lasted for 4 weeks. We observed the physiological state of the mice and weighed them.

Subsequently, 7-week-old SPF female nude mice were used for subcutaneous tumorigenesis experiments and divided into two groups. Each mouse was inoculated with 1 × 10^6^ BT549 cells. Ten days later, mice in the experimental group were given i.p. of BC (10 mg/kg/d), while mice in the control group received an equal volume of normal saline. The treatment lasted 4 weeks. Tumor volume was measured, and the weight change of mice was recorded. All mice were sacrificed, the tumors were surgically removed and then weighed, and the kidney, liver, heart, spleen, and lung were collected and fixed in 4% paraformaldehyde for Pathological analysis.

All experiments were conducted with the approval of the Experimental Animal Management Committee of Sichuan University and under the supervision of the Ethical Review of Animal Experimentation Welfare (No. K2024013).

#### 2.2.10 Hematoxylin-eosin (H&E) staining

For H&E staining, paraffin-embedded mice organ sections were then dehydrated with different concentrations of Dimethyl benzene and ethanol, followed by incubation with 5% hematoxylin solution for 5 min. After washes, the sections were stained in 0.1% HCl-ethanol for 1–3 s and then soak in water for 5 min. Finally, after a 5-min reaction with eosin solution, dehydration and sealing with neutral gum, the sections were examined microscopically and photographed.

### 2.3 Statistical analysis

All experiments involving BT549 cells were repeated three times. All statistical analyses were performed using R software and GraphPad prism 8.0. Statistical significance was assessed by t-test and one-way ANOVA: *P < 0.05; **P < 0.01; ***P < 0.001; ****P < 0.0001.

## 3 Results

### 3.1 Analysis of active compounds, potential targets and pathways based on network pharmacology

#### 3.1.1 BC was the main active compound and there were 89 intersection targets and multiple signaling pathways for the treatment of TNBC by BC

We used TCMSP database to predict the active compounds of Baiji, and a total of nine active compounds were retrieved (Table 1). After the pre-drug activity screening, we found that BC had a better inhibitory effect on TNBC cells.

**TABLE 1:** Basic information of the active components of Baiji.

Molecule name	Mol ID	Molecule weight	OB (%)	DL
Bletlol A	MOL005770	466.57	54.43	0.55
Orchidble	MOL005755	348.42	54.18	0.55
Blespirol	MOL005773	398.43	43.74	0.86
2,3,4,7-tetramethoxyphenanthrene	MOL005756	298.36	39.09	0.29
SCHEMBL1975481	MOL005761	348.42	37.98	0.55
Blestriarene C	MOL005776	478.52	35.22	0.67
3,7-dihydroxy-2,4-dimethoxyphenanthrene-3-O-glucoside	MOL005766	428.52	31.46	0.78
CHEMBL365485	MOL005768	348.42	30.54	0.55
2,7-dihydroxy-4-methoxyphenanthrene-2,7-O-diglucoside	MOL005759	550.56	30.22	0.74

Then, a total of 126 and 65 targets were collected in different drug databases, and 186 drug candidate targets were finally obtained.

1,597 and 1,553 disease genes were searched, and 2,201 disease genes were obtained by combining the disease targets from different databases and deleting the duplicates.

Venny was used to screen out a total of 89 intersection targets of BC and TNBC-related targets, as shown in Figure 1B.

A total of 86 pathways (*P* < 0.05) were obtained from KEGG enrichment analysis, mainly involving microRNAs in cancer, MAPK signaling pathway and PI3K-Akt signaling pathway, etc. The most significant enriched 20 pathways in KEGG analysis were shown in Figure 1C.

A total of 348 GO items (*P* < 0.05) were obtained, including 220 BP, 56 CC, and 74 MF. The first 10 items of enrichment results were visualized according to *p* value, as shown in Figure 1D.

#### 3.1.2 PPI network construction and module analysis suggested the MAPK signaling pathway and cell cycle

To further reveal the potential relationships between the encoded proteins, the PPI network of the candidate targets was screened by STRING, including 89 nodes and 454 edges. We used MCODE to perform module analysis to detect critical clustering modules. Four modules were retrieved from the PPI network constructed. Module 1 included 13 nodes and 84 edges with a cluster score of 7.000 (Figure 1E). Module 2, with 19 nodes and 82 edges, had a score of 4.556 (Figure 1F). Module 3 including 9 nodes and 36 edges had a score of 4.500 (Figure 1G). Module 4 included 3 nodes and 6 edges having a score of 3.000 (Figure 1H).

As shown in Figures 1E–H, the results illustrated that module 1 was enriched in ERBB signaling pathway, and ERK1 and ERK2 cascade; module 2 focused on MAPK signaling pathway; module 3 involved DNA damage and cell cycle; and module 4 was related to cell adhesion.

#### 3.1.3 HSP90AA1 and PTGS2 were selected as hub genes for subsequent studies

**FIGURE 2:**
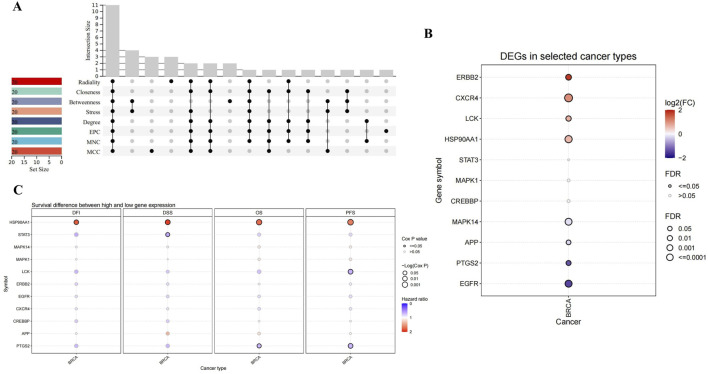
Upset plot and analysis of hub genes. **(A)** Eight algorithms have screened out 11 overlapping hub genes. **(B)** The expression in BRCA of hub genes. **(C)** Survival difference between high and low gene expression in BRCA.

Through the eight algorithms of cytoHubba, we have calculated the top 20 hub genes. After taking the intersection of the upset plot, we found 11 common hub genes, including EGFR, HSP90AA1, STAT3, ERBB2, MAPK1, PTGS2, MAPK14, CXCR4, LCK, APP, and CREBBP (Figure 2A).

As shown in Figure 2B, CXCR4, ERBB2, LCK and HSP90AA1 expression were upregulated, while EGFR, PTGS2, APP and MAPK14 expression downregulated in BRCA. Considering the four survival indicators (DFI, DSS, OS and PFS), the expression of HSP90AA1 and PTGS2 had a significant effect on survival (*P* < 0.05) (Figure 2C). We chose HSP90AA1 and PTGS2 as hub genes for subsequent studies.

#### 3.1.4 MiR-146a-5p was selected as the key microRNA

**FIGURE 3:**
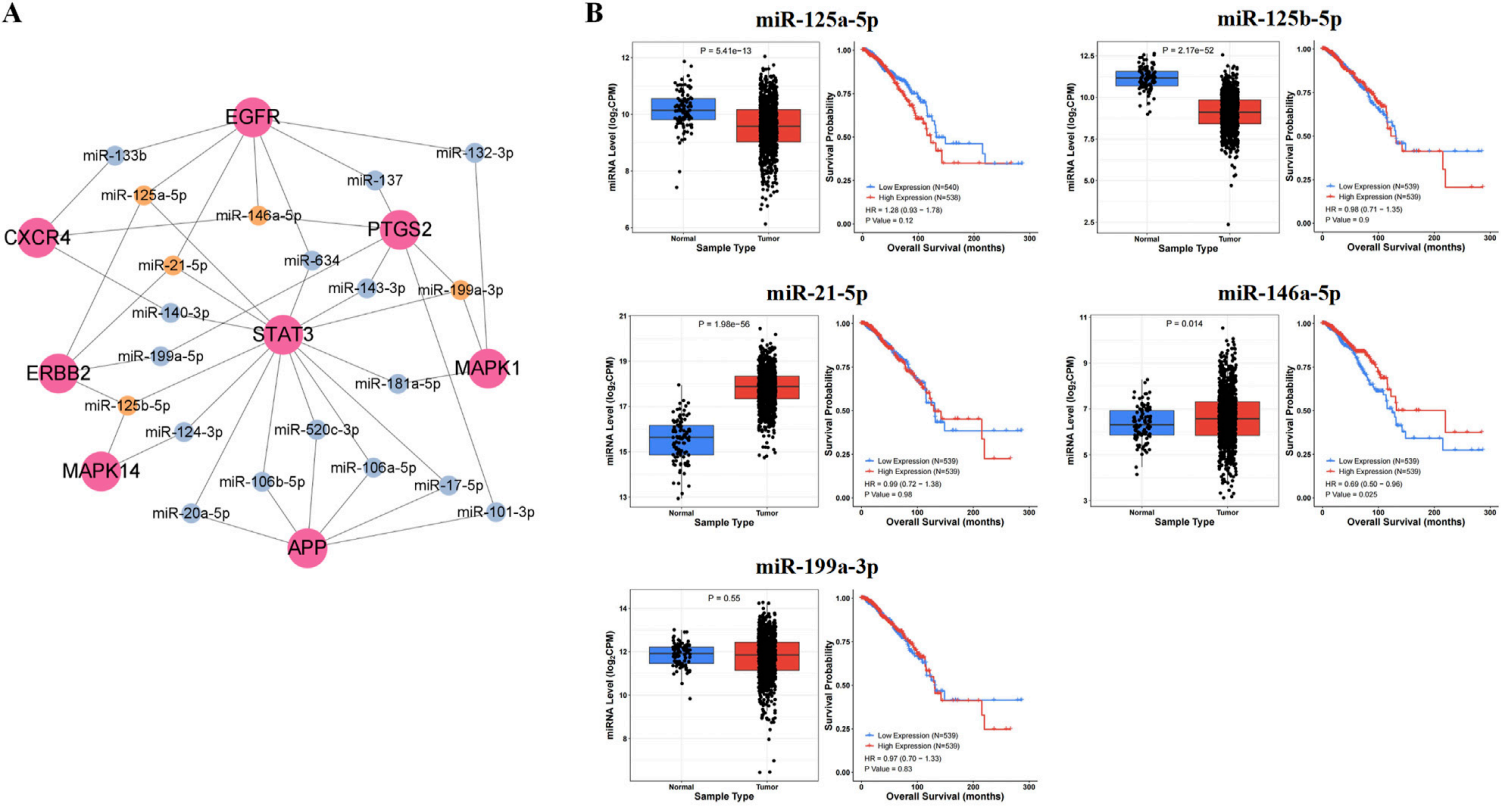
Prediction results of key microRNAs. **(A)** The interaction network diagram of “core target-microRNA.” **(B)** The expression levels and prognostic value in BRCA of key microRNAs.

In the miRTarBase database, we found microRNAs targeting core proteins, including 7 targeting APP, 11 targeting CXCR4, 31 targeting EGFR, 10 targeting ERBB2, 1 targeting HSP90AA1, 16 targeting MAPK1, 6 targeting MAPK14, 10 targeting PTGS2, and 42 targeting STAT3. In addition, we constructed the interaction network diagram of “core target-microRNA” and screened the five ten microRNAs (miR-125a-5p, miR-125b-5p, miR-21-5p, miR-146a-5p, miR-199a-3p) in Cytoscape 3.9.1 (Figure 3A).

After analyzing the expression and survival curves of microRNAs in BRCA, we found that miR-21-5p and miR-146a-5p were overexpressed, while miR-125a-5p and miR-125b-5p expression decreased. Results from the Kaplan-Meier survival analysis showed that miR-146a-5p was associated with survival in breast cancer patients (*P* < 0.05) (Figure 3B).

#### 3.1.5 High expression of HSP90AA1 and low expression of PTGS2 in BRCA

**FIGURE 4:**
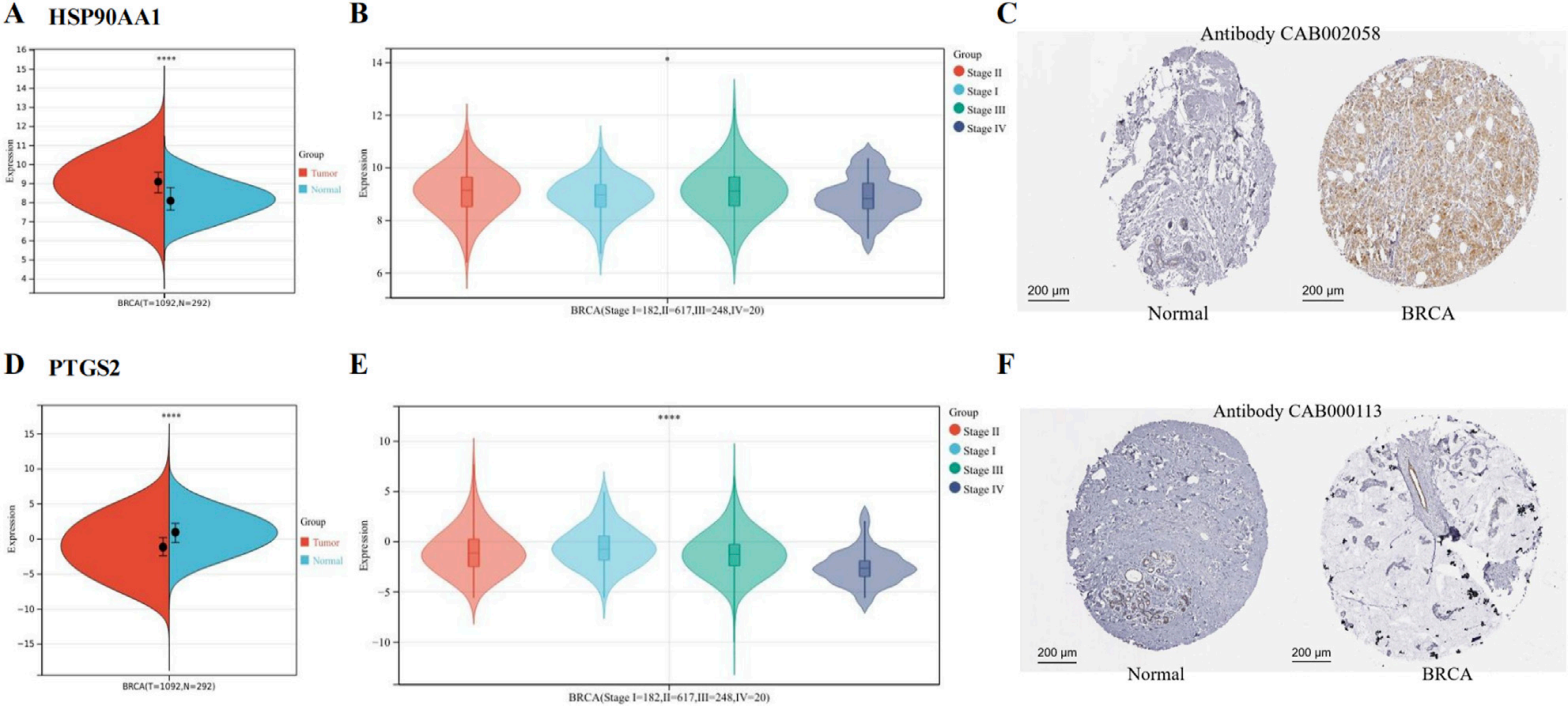
Expression of hub genes in BCRA from TCGA and GTEx data. **(A)** HSP90AA1 was upregulated in BRCA. **(B)** The expression of HSP90AA1 was associated with the stage of BRCA. **(C)** The protein expression of HSP90AA1 in BRCA. **(D)** PTGS2 was downregulated in BRCA. **(E)** The expression of PTGS2 was associated with the stage of BRCA. **(F)** The protein expression of PTGS2. (*P < 0.05 and ****P < 0.0001 versus control group).

We found that HSP90AA1 was upregulated (Figure 4A), and its expression was associated with the stage of BRCA (Figure 4B). The IHC results provided by the HPA database further confirmed that HSP90AA1 was highly expressed (Figure 4C). In contrast, PTGS2 expression was reduced (Figure 4D), and its expression was also associated with the stage of BRCA (Figure 4E). The IHC results further confirmed that PTGS2 was low expressed in BRCA patients (Figure 4F).

Table 2 showed the content of the survival data for both HSP90AA1 and PTGS2.

**TABLE 2:** The survival data content of HSP90AA1 and PTGS2.

Cancer	Gene	Survival type	Hazard ratio	Cox P value	Logrank P value	Higher risk of death
BRCA	HSP90AA1	OS	1.73	9.15e^−4^	7.91e^−4^	Higher
		PFS	1.64	4.29e^−4^	3.75e^−4^	
		DSS	1.90	4.03e^−3^	3.47e^−3^	
		DFI	1.79	9.25e^−3^	8.31e^−3^	
	PTGS2	OS	0.70	0.03	0.03	Lower
		PFS	0.68	6.50e^−3^	6.14e^−3^	
		DSS	0.69	0.09	0.08	
		DFI	0.69	0.10	0.09	

#### 3.1.6 Mutations and promoter methylation were found in both HSP90AA1 and PTGS2

**FIGURE 5:**
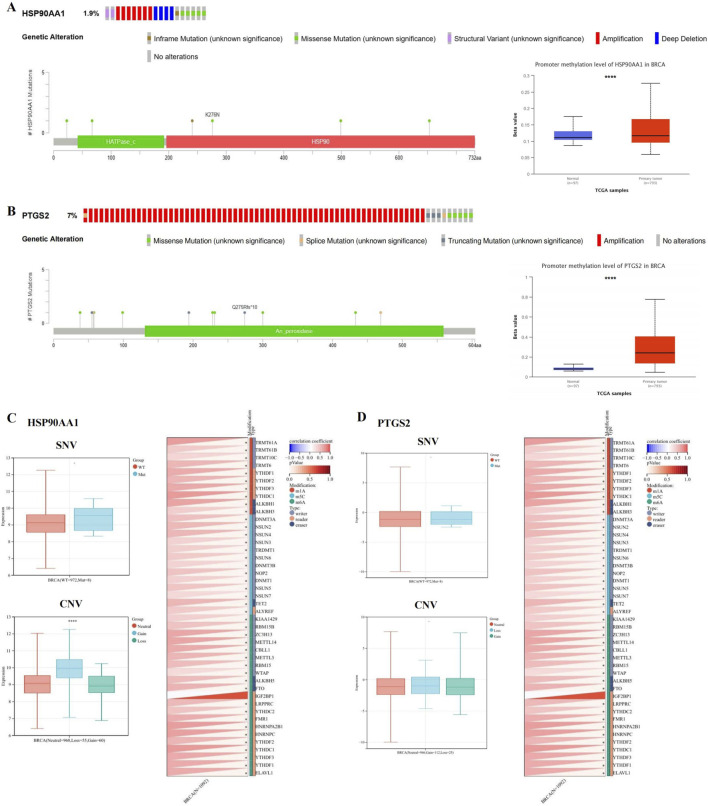
HSP90AA1 and PTGS2 gene mutation and methylation in BRCA. **(A, B)** The alteration frequency, mutation types, mutation sites, and promoter methylation level of HSP90AA1 and PTGS2. **(C)** The relationship between SNV, CNV and HSP90AA1 expression, and the correlation between HSP90AA1 and the expression of 44 marker genes of RNA modification. **(D)** The relationship between SNV, CNV and PTGS2 expression, and the correlation between PTGS2 and the expression of 44 marker genes of RNA modification. (*P < 0.05, ****P < 0.0001 versus control group).

As shown in Figures 5A, B, mutation of HSP90AA1 and PTGS2 were observed in BRCA patients. Among 996 sequenced BRCA patients, genetic variants were found in 19 and 73 patients, respectively, with mutation rates of 1.9% and 7%.

We compared the methylation levels of HSP90AA1 and PTGS2 between normal and BCRA tissues, and found that the methylation levels of HSP90AA1 and PTGS2 promoters were significantly increased (Figures 5A, B), indicating that the transcriptional expression may be affected by the alterations of promoter methylation. In addition, the expression of HSP90AA1 was significantly associated with CNV, but not with SNV. Finally, the HSP90AA1 and PTGS2 genes were positively correlated with the marker genes of most RNA modification (Figures 5C, D).

### 3.2 Experimental validation

#### 3.2.1 BC had significant inhibitory effect on BT549 cell proliferation and migration

**FIGURE 6:**
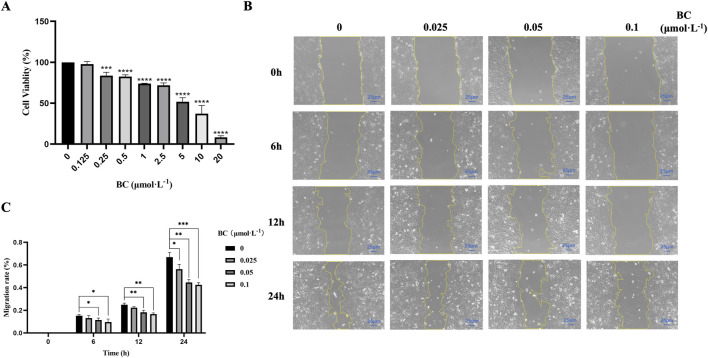
BC has significant inhibition effect on BT549 cell proliferation and migration. **(A)** Cell viability of BT549 cells upon BC treatment. **(B)** Microscope image of wound healing assay at different time points. **(C)** BC has significant inhibition effect on BT549 cell migration. (*P < 0.05, **P < 0.01, ***P < 0.001, ****P < 0.0001 versus control group).

The results of CCK-8 assay showed that BC at different concentrations significantly decreased BT549 cell viability following 24 h treatment, and the cell viability rate gradually decreased with the increase of concentration, indicating that BC had significant inhibition effect on BT549 cells (Figure 6A). The IC_50_ value had been calculated to be 7.671 μmol. L^−1^.

Based on the results of the CCK8 assay, we selected the concentrations (below 0.125 μmol. L^−1^) to investigate the effect of BC on BT549 cell migration. As shown in Figures 6B, C, we found that BC above 0.05 μmol. L^−1^ can exert migration inhibition after 6 h, and after 24 h, lower concentrations of BC can also significantly inhibit the migration of BT549 cells.

#### 3.2.2 BC induced apoptosis in BT549 cells

**FIGURE 7:**
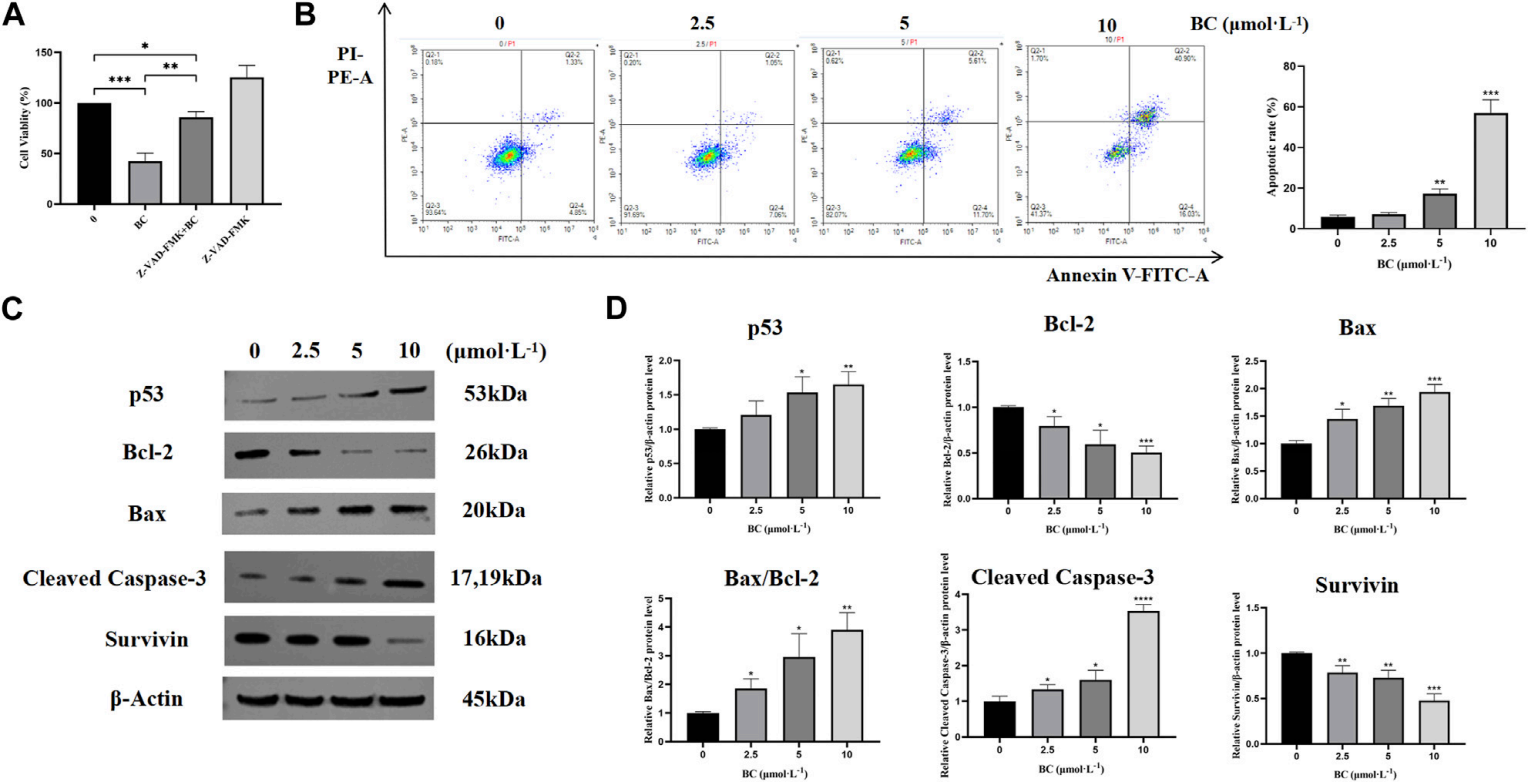
BC induced apoptosis in BT549 cells. **(A)** Cell viability of BT549 cells after combination with Z-VAD-FMK. **(B)** Results of Annexin V-FITC/PI apoptosis assay after BC treatment. **(C)** Images of apoptosis-related protein expression bands. **(D)** Statistical results of apoptosis-related protein expression. (*P < 0.05, **P < 0.01, ***P < 0.001, ****P < 0.0001 versus control group).

We found that after apoptosis inhibition, the survival rate of BT549 cells was significantly increased after the action of BC (Figure 7A), which indicated that BC induced the death of BT549 cells through inducing apoptosis. And after BC treatment, the proportions of both early and late apoptotic cells were increased, suggesting that BC could induce apoptosis in BT549 cells in a dose-dependent manner (Figure 7B).

To further investigate the mechanism of apoptosis induction in BT549 cells by BC, we detected the protein expression levels of bcl-2, bax, cleaved caspase-3, and survivin. BC could significantly promote the expression of apoptotic protein bax and cleaved caspase-3, while decreasing the expression of anti-apoptotic proteins bcl-2 and survivin, indicating that BC can induce apoptosis in BT549 cells (Figures 7C, D).

#### 3.2.3 BC caused S-phase cycle arrest in BT549 cells

**FIGURE 8:**
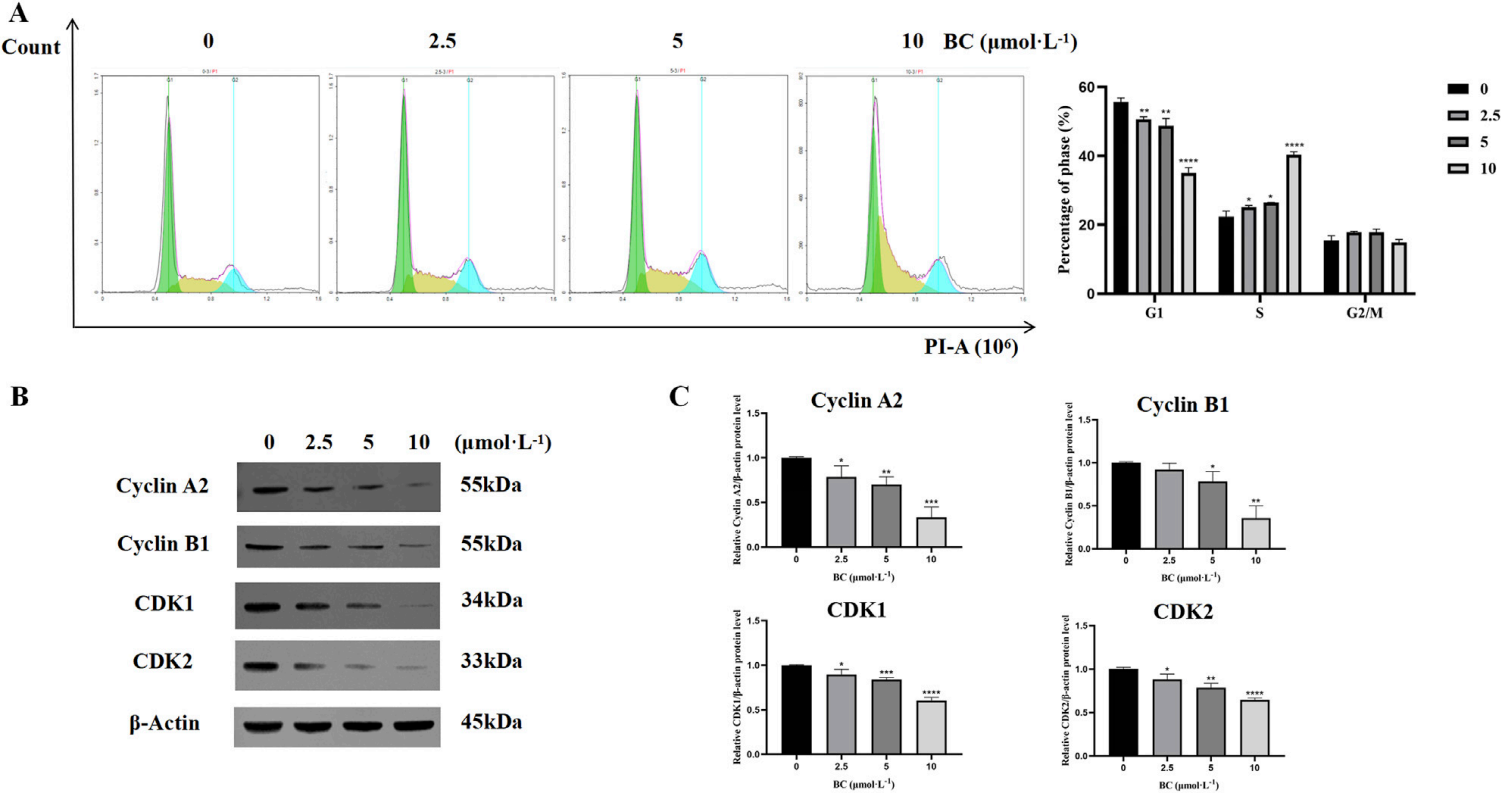
BC caused BT549 cell cycle arrest. **(A)** Cell cycle assay results after BC treatment. **(B)** Images of cell cycle-related protein expression bands. **(C)** Statistical results of cell cycle-related protein expression. (*P < 0.05, **P < 0.01, ***P < 0.001, ****P < 0.0001 versus control group).

After treatment with BC, the proportion of cells in the G1 phase was decreased, while the proportion of cells in the S phase was significantly increased, showing that BC blocked the BT549 cell cycle mainly in the S phase (Figure 8A).

We evaluated the expression of cell cycle-related proteins cyclin A2, and CDK2 and found that the expression of all four proteins was significantly reduced and that BC could induce S-phase cycle arrest in BT549 cells in a dose-dependent manner (Figures 8B, C).

#### 3.2.4 BC suppressed the Ras/ERK/c-Fos signaling pathway

**FIGURE 9:**
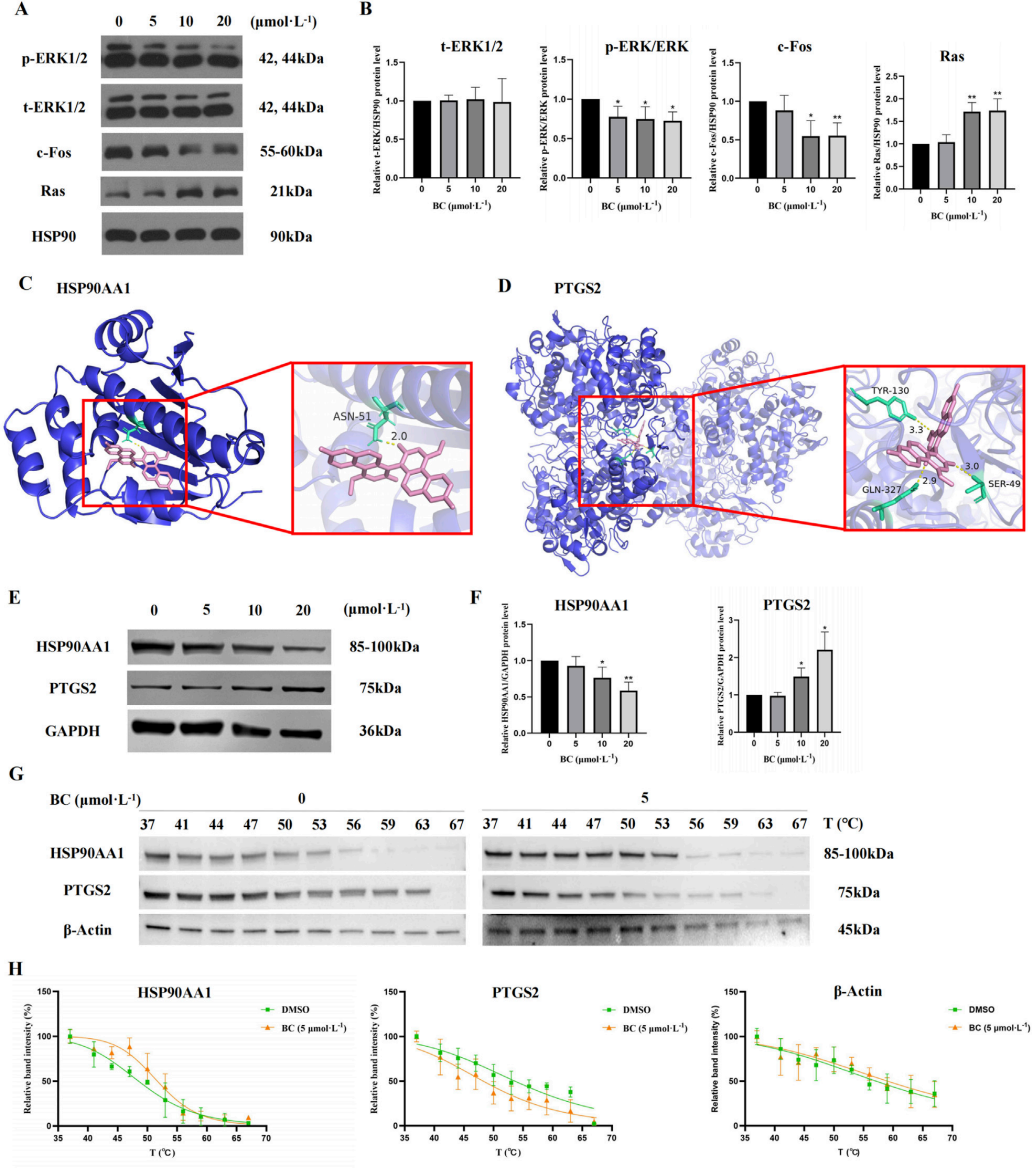
Effect of BC on the expression of diverse proteins in BT549 cells. **(A)** Image of MAPK signaling pathway-related protein expression bands. **(B)** The expression of MAPK signaling pathway-related proteins after BC treatment of BT549 cells. (*P < 0.05, **P < 0.01 versus control group). **(C)** Molecular docking diagram of BC and HSP90AA1 protein. **(D)** Molecular docking diagram of BC and PTGS21 protein. **(E)** Image of hub protein expression bands. **(F)** The expression of hub proteins after BC treatment of BT549 cells. **(G)** Images of HSP90AA1, PTGS2, and β-Actin protein expression bands of the cellular thermal shift assay. **(H)** Statistical results of HSP90AA1, PTGS2, and β-Actin protein expression bands of the cellular thermal shift assay. (*P < 0.05, **P < 0.01 versus control group).

We examined the MAPK signaling pathway-related proteins of BT549 cells exposed to BC. The results demonstrated that BC could promote the expression of Ras, promote the phosphorylation of ERK1/2, and inhibit the expression of c-Fos (Figures 9A, B), indicating that BC acts by regulating the Ras/ERK/c-Fos signaling pathway.

#### 3.2.5 BC had strong binding activity with HSP90AA1 and PTGS2 and regulated their expression

It is generally believed that when the binding energy is less than −7.0 kcal. mol^−1^, this compound has a strong binding activity with the core target protein. The best binding energy data between HSP90AA1 or PTGS2 and BC were −8.3 kcal. mol^−1^ or -10.2 kcal. mol^−1^, indicating that BC had strong binding activity with HSP90AA1 and PTGS2 protein.

Figures 9C, D showed the docking diagram of BC with HSP90AA1 and PTGS2 protein molecules.

For hub proteins, BC can downregulate the expression of HSP90AA1 and upregulate the expression of PTGS2 (Figures 9E, F).

Furthermore, as illustrated in Figures 9G, H, the apparent melting curves of HSP90AA1 exhibited a notable shift to the right following BC treatment, suggesting that the stability of HSP90AA1 protein was enhanced, and the amount of soluble proteins was increased under identical temperature conditions. Conversely, PTGS2 displayed a significant shift to the left, indicating that the stability of PTGS2 protein was diminished following BC treatment. The T_m_ values of HSP90AA1 before and after drug addition were 48.51°C and 51.67°C, and those of PTGS2 were 53.60°C and 48.25°C.

#### 3.2.6 BC significantly inhibited TNBC and had a high safety profile

**FIGURE 10:**
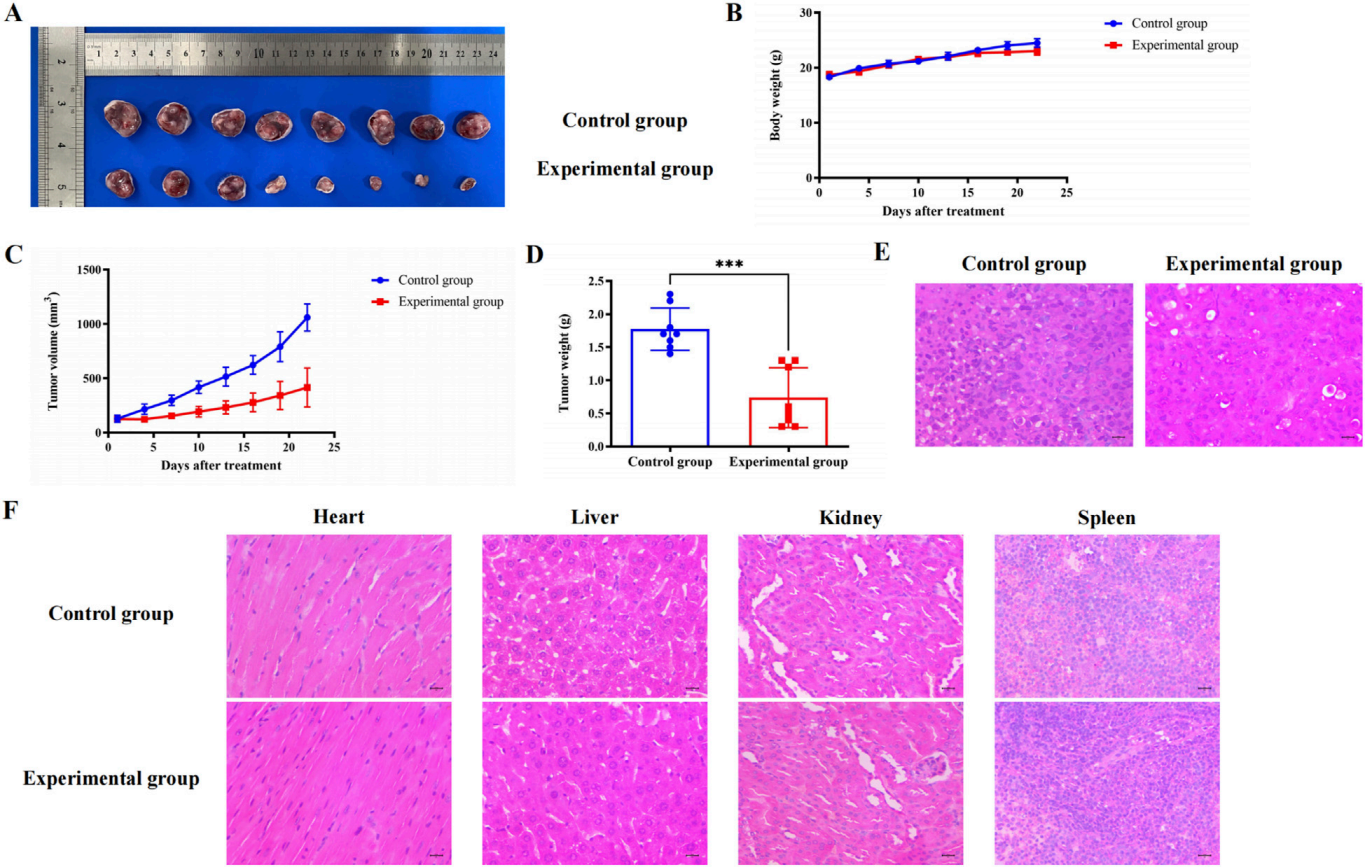
Effect of BC on subcutaneous tumors of TNBC in mice. **(A)** Pictures of TNBC tumors treated with BC. **(B)** BC did not significantly reduce the body weight of mice that constructed subcutaneous tumors. **(C)** Change in weight of TNBC tumors after treatment with BC. **(D)** Volume change in TNBC tumors after treatment with BC. **(E)** H&E staining of TNBC tumors after receiving BC treatment (40 × ) **(F)** Representative graph of H&E staining of heart, liver, kidney, and spleen after treatment with BC (40 × ).

In the early stage, we found that BC had no significant effect on the survival and body weight of nude mice. Subsequently, we constructed a subcutaneous tumor model of TNBC and found that BC can significantly inhibit the volume and weight of TNBC tumors without significantly reducing the body weight of mice (Figures 10A–D). The H&E staining results of TNBC tumors in nude mice also suggested that BC significantly inhibited the growth of TNBC tumors (Figure 10E). Moreover, H&E staining of the heart, liver, spleen, lung and kidney tissues of mice showed no obvious pathological changes in the organ (Figure 10F), indicating that BC has a significant inhibitory effect on TNBC and has a high safety profile, and is a potential therapeutic drug for TNBC.

## 4 Discussion

There are already some treatments for breast cancer, such as surgery, radiation therapy, chemotherapy, hormone therapy, targeted therapy, etc. (Maughan et al., 2010). However, TNBC is insensitive to endocrine therapy and targeted therapy because there is no corresponding target to be found, and the currently available treatments are relatively limited (An et al., 2021).

An increasing number of studies have shown that the discovery of drug candidates from traditional Chinese medicine has become a trend in new drug research (Han et al., 2024). At present, many ingredients extracted from traditional Chinese medicine have been included in breast cancer trials and achieved certain results (Chen et al., 2022; Du et al., 2021; Wang et al., 2019). However, there are still some problems: basic research is still relatively insufficient, and the mechanism of action is still not clear.

Based on network pharmacology analysis, the potential mechanism of action of BC extracted from traditional Chinese herbal medicine in TNBC treatment was systematically analyzed, and related *in vivo* and *in vitro* experiments were designed for verification.

The high expression of HSP90AA1 and the low expression of PTGS2 were both associated with different stages of BRCA, suggesting that they may act as potential makers for judging different stages of TNBC. Furthermore, we investigated the mutation and methylation of HSP90AA1 and PTGS2. Interestingly, both HSP90AA1 and PTGS2 had elevated promoter methylation levels, but this change promoted HSP90AA1 expression and inhibited PTGS2 expression. It is generally accepted that promoter DNA methylation is associated with transcriptional inhibition (Curradi et al., 2002; Jones, 2012). While this association has been increasingly questioned, promoter hypermethylation is also thought to be associated with high transcriptional activity, particularly in many malignancies (Smith et al., 2020). We hypothesize that in TNBC, promoter hypermethylation either induces or inhibits hub gene expression in different ways, but this requires further evidence.

Apoptosis, as a mode of programmed cell death, plays an indispensable and critical role in the development, treatment and prognosis of TNBC (Parton et al., 2001). Proteins encoded by the bcl-2 gene family are critical for the regulation of apoptosis and are overexpressed in approximately 80% of breast cancer patients (Krajewski et al., 1999). Caspase is a novel biomarker associated with apoptosis and is an important intracellular effector in the formation of apoptotic cells. We found that BC can significantly inhibit bcl-2 and survivin protein expression while promoting bax and cleaved caspase-3 protein expression, which has a better apoptosis-inducing effect on TNBC cells.

As a tumor suppressor, p53 plays a central role in apoptosis, cell cycle, and DNA damage response (Ferreira et al., 1999). A variety of current clinical breast cancer treatments induce apoptosis through p53-dependent pathways (Bergh, 1999). BC can promote the expression level of p53, which in turn promotes apoptosis and blocks the cell cycle through the p53 signaling pathway. Furthermore, the expression of cell cycle-related proteins cyclin A2, cyclin B1, CDK1, and CDK2 was decreased after treatment with BC. The binding of cyclin A and CDK2 was inhibited, and the cell cycle of TNBC cells was blocked in the S phase.

Molecular docking results showed that BC had good affinity with the key targets HSP90AA1 and PTGS2, and the conformation of the binding site was stable and the binding ability was strong.

**FIGURE 11:**
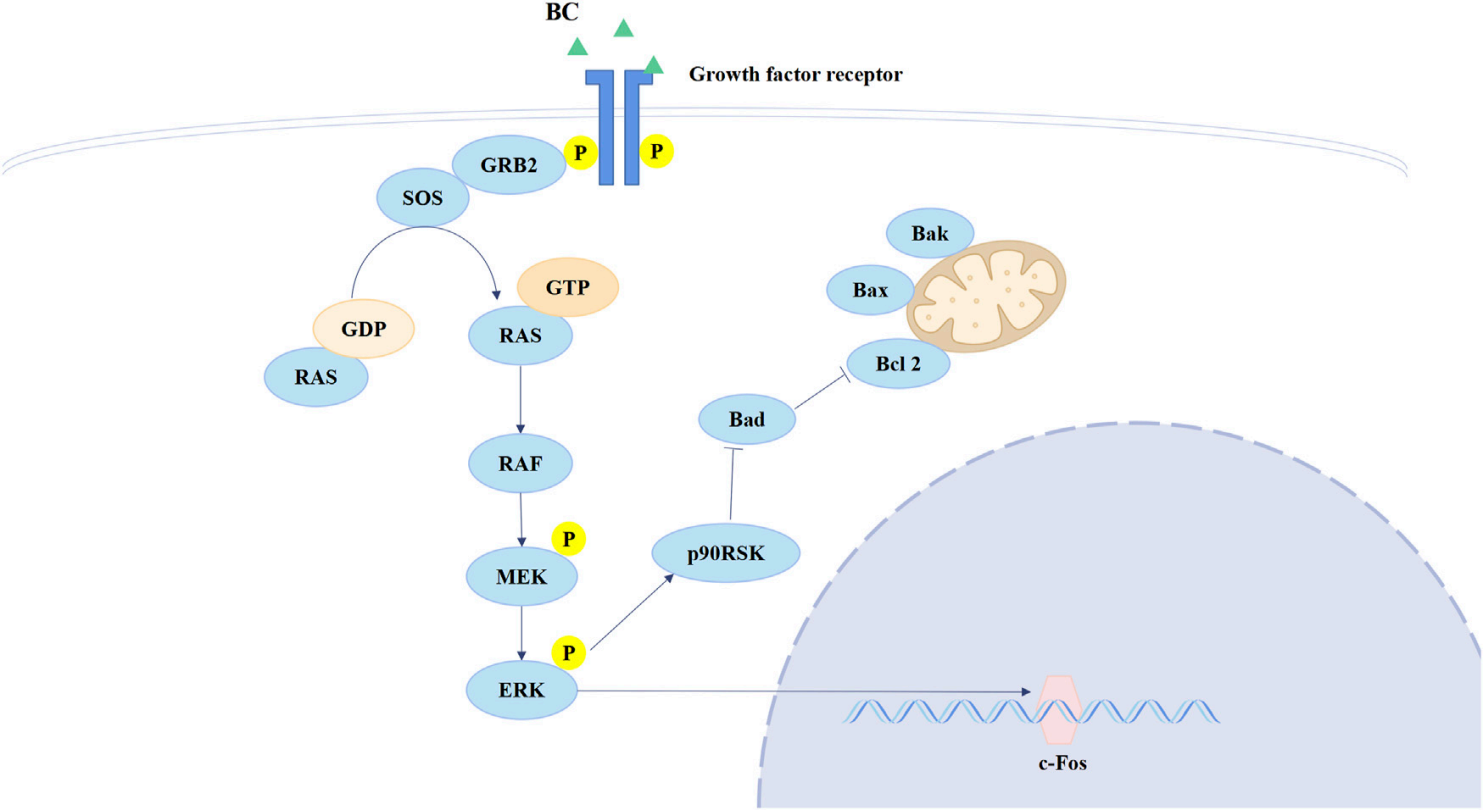
Mechanism of Ras/ERK/c-Fos signaling pathway.

Ras is the most frequently mutated gene family in tumors and is activated at the membrane downstream of growth factor receptors, including members of the EGFR family, which regulates both the MAPK and PI3K signaling pathways (Moore et al., 2020). ERK belongs to the MAPK family, the activation of which could directly phosphorylate c-Fos, regulating cell proliferation and cell differentiation (Lavoie et al., 2020). Currently, therapies that target RAS directly or in combination with other therapies are potential treatments for RAS-mutated tumors (Downward, 2003). The Ras/ERK/c-Fos pathway is shown in Figure 11. BC can increase Ras expression but inhibit p-ERK and c-Fos expression, and we speculate that BC hinders the process of RAS conversion to RAF, allowing RAS to accumulate in cells, thereby inhibiting the Ras/ERK/c-Fos pathway. This suggests that BC may be an inhibitor of RAS and provides new hope for RAS-targeted therapy of TNBC. Moreover, a study showed that the inhibition of HSP90 significantly suppressed cell proliferation and induced cell apoptosis in lung cancer via downregulating AKT1-ERK pathway, where the inhibition of HSP90 decreased p-ERK expression (Niu et al., 2021); Mesenchymal stem cell-derived exosomes could relieve hypoxic pulmonary hypertension by suppressing the Hsp90AA1/ERK/pERK pathway, in which the decrease of p-ERK could also be observed (Deng et al., 2024).

We used the subcutaneous tumorigenic model to verify the inhibitory effect of BC in mice. Fortunately, we found that BC had no significant effect on rat body weight while inhibiting TNBC tumor growth, indicating that BC had a good therapeutic effect and low toxicity on TNBC.

## 5 Conclusion

To sum up, it was the first time we explored the underlying mechanism of the inhibition of Baiji and BC on TNBC. Through network pharmacology analysis, we screened out BC as the main active ingredient and predicted that BC may be involved in MAPK, PI3K-Akt and Ras signaling pathway, and regulate microRNAs expression, affecting apoptosis and cell cycle. Among them, HSP90AA1 and PTGS2 are core genes of BC for TNBC treatment. Molecular docking verified that BC had a strong binding ability to HSP90AA1 and PTGS2. Cell experiments showed that BC could inhibit the Ras/ERK/c-Fos signaling pathway while downregulating HSP90AA1 expression and upregulating PTGS2 expression, thereby promoting apoptosis, causing S-phase cycle arrest, and inhibiting the proliferation and migration of BT549 cells. Experiments in mice have shown that BC can inhibit the growth of TNBC tumors and has a high safety profile. These results provide a basis for further biological research and clinical application of BC in TNBC treatment. However, other biological pathways and mechanisms involved in the treatment of BC need to be further validated.

## Data Availability

The datasets presented in this study can be found in online repositories. The names of the repository/repositories and accession number(s) can be found in the article/Supplementary Material.
