# Supplementary material for: Blestriarene C exerts an inhibitory effect on triple-negative breast cancer through multiple signaling pathways
Source: Front Pharmacol. 2024 Oct 22;15:1434812. doi: 10.3389/fphar.2024.1434812 (PMC11534688; doi:10.3389/fphar.2024.1434812)
Supplement: Supplementary file 1 [file Table1.docx]

**Supplementary Table 1.** The databases and their web addresses used in this article.

| **Methods** | **Databases** | **Web addresses** |
| --- | --- | --- |
| Prediction of active compounds and candidate targets | TCMSP database | https://old.tcmsp-e.com/tcmsp.php |
|  | SuperPred database | https://prediction.charite.de/ |
|  | SwissTargetPrediction database | http://swisstargetprediction.ch/ |
|  | DisGeNet database | https://www.disgenet.org/ |
|  | GeneCards database | https://www.genecards.org/ |
|  | Venny 2.1.0 | https://bioinfogp.cnb.csic.es/tools/venny/ |
| PPI network construction | String database | https://cn.string-db.org/ |
|  | GeneMANIA database | http://genemania.org/ |
| Screening and analysis of hub genes | GSCA database | http://bioinfo.life.hust.edu.cn/GSCA/#/ |
|  | miRTarBase database | https://mirtarbase.cuhk.edu.cn/ |
|  | CancerMIRHome database | http://bioinfo.jialab-ucr.org/ |
|  | HPA database | https://www.proteinatlas.org/ |
|  | cBioPortal database | https://www.cbioportal.org/ |
|  | Ualcan database | http://ualcan.path.uab.edu/index.html |
| Molecular docking | PubChem database | https://pubchem.ncbi.nlm.nih.gov/ |
|  | PDB database | http://www.rcsb.org/ |
